# Early rhythmicity in the fetal suprachiasmatic nuclei in response to maternal signals detected by omics approach

**DOI:** 10.1371/journal.pbio.3001637

**Published:** 2022-05-24

**Authors:** Philipp Greiner, Pavel Houdek, Martin Sládek, Alena Sumová

**Affiliations:** Laboratory of Biological Rhythms, Institute of Physiology of the Czech Academy of Sciences, Prague, Czech Republic; Charité - Universitätsmedizin Berlin, GERMANY

## Abstract

The suprachiasmatic nuclei (SCN) of the hypothalamus harbor the central clock of the circadian system, which gradually matures during the perinatal period. In this study, time-resolved transcriptomic and proteomic approaches were used to describe fetal SCN tissue-level rhythms before rhythms in clock gene expression develop. Pregnant rats were maintained in constant darkness and had intact SCN, or their SCN were lesioned and behavioral rhythm was imposed by temporal restriction of food availability. Model-selecting tools dryR and CompareRhythms identified sets of genes in the fetal SCN that were rhythmic in the absence of the fetal canonical clock. Subsets of rhythmically expressed genes were assigned to groups of fetuses from mothers with either intact or lesioned SCN, or both groups. Enrichment analysis for GO terms and signaling pathways revealed that neurodevelopment and cell-to-cell signaling were significantly enriched within the subsets of genes that were rhythmic in response to distinct maternal signals. The findings discovered a previously unexpected breadth of rhythmicity in the fetal SCN at a developmental stage when the canonical clock has not yet developed at the tissue level and thus likely represents responses to rhythmic maternal signals.

## Abstract 2

Circadian gene and protein expression profiling reveals that fetal rat suprachiasmatic nuclei respond rhythmically to distinct maternal signals before rhythmicity develops in the expression of canonical clock genes.

## Introduction

Most organisms are exposed to conditions that vary with a periodicity of solar days. Therefore, they organize intrinsic processes rhythmically according to expected changes in the environment. The ability to anticipate these variations represents a crucial advantage, and, as a result, biological processes not only passively follow changes in external conditions but are driven by endogenous clocks [1]. In the absence of external cues, these clocks run with periods close to the solar day (circadian from Latin circa diem), but when exposed to the day–night cycle, they entrain precisely with the 24-hour solar cycle [2,3]. Rhythmic circadian signals are generated at the cellular level via autonomously driven cycles in the expression of clock genes and their proteins, which play a regulatory role in the core clock mechanism [4]. In the mammalian body, these cellular clocks are organized hierarchically, with a central clock at the top, due to its exclusivity in sensing external light and its ability to control systemic rhythms that entrain other clocks in the body. This central clock is located in the suprachiasmatic nuclei (SCN) of the hypothalamus [5] and consists of thousands of cellular oscillators interconnected by a network of synapses and organized into functionally specialized subpopulations [6], providing the SCN with the properties of the master clock in the body.

Previous research in laboratory rodents clearly indicated that morphologically the SCN develops gradually from the late gestational to the early postnatal period [7,8]. The gestational period takes 18 to 19 days in mice and 20 to 21 days in rats, and neurogenesis of the SCN is completed around embryonic day (E)14 and E15, respectively [9,10]. However, adult-like morphological organization is not achieved prenatally, as the SCN at this stage still lacks the synapses [11] that are required for communication between individual cellular oscillators [12]. Synaptogenesis accelerates only after birth and progresses until the 10th postnatal day [9]. Importantly, this process proceeds in close correlation with the gradual increase in the robustness of SCN oscillation as measured by amplitudes of clock gene expression and protein rhythms at the cell population level [13–16]. Attaining the mature state is conditional for the SCN to fulfill the role of the central clock in the circadian system.

The precise timing of the appearance of endogenous rhythmicity in the SCN remains a matter of debate, mainly because it depends heavily on the experimental approach of its detection [17], as well on whether single-cell or cell-population rhythms are required as measure of the “presence of the clock.” Detailed analyses of the timing of the first appearance of rhythms in PER2-driven bioluminescence at the cellular level in fetal SCN of *mPer2*^*Luc*^ mouse detected first rhythms in a few individual cells of unidentified origin already at E14.5 to E15.5. However, more cells became rhythmic as fetal development progressed later on [18]. It corresponded with previously observed later gradual development of the in vitro cell population bioluminescence rhythmicity in the mouse SCN [19–21]. Nevertheless, the relevance of this approach for the purpose of detecting the initiation of rhythmicity has been questioned, as placing rat SCN tissue from arrhythmic animals into culture induced rhythmicity [22]. Indeed, cell population clock gene expression rhythms of very low amplitude in the fetal rat SCN sampled from dams around the clock in vivo could be first detected by sensitive quantitative reverse transcription PCR (RT-qPCR) technique only at E20 to E21 [23]. The intriguing question remains as to how synchrony between individual SCN cells, which is required for the detection of tissue-level rhythms, is attained in the immature SCN still lacking web of synapses.

Considering all the data, it can be generalized for both rodent species that very shallow clock gene expression rhythms can be first detected at the SCN tissue level only about 1 to 2 days before birth. Additionally, in the fetal rat, SCN rhythms have been demonstrated in glucose utilization at E19 to E21 [24,25], arginine vasopressin (*Avp*) expression at E20 to E21 [14,26], firing rate at E22 [27], and *c-fos* expression associated with neuronal activity at E19 [23,28]. We previously hypothesized [29] that rhythmic environment provided by maternal signals during the perinatal period (reviewed in [9,30,31]) may drive rhythmicity in cellular processes in the fetal SCN before spontaneous oscillatory mechanisms within individual cells are synchronized resulting in cell population rhythmicity. We suggest that maternal signals may conduct these cellular rhythms in the absence of intercellular synchrony between developing oscillators in the fetal SCN. Consistent with this, maternal signals reset the phase of the developing SCN clock, as documented by evidence that shifting the maternal SCN clock during gestation reprograms the phase of developing fetal clock [32] and fetal SCN responds to their disruption even at a stage when SCN tissue-level rhythms in clock gene expression were not yet developed [28].

The extent of rhythmicity in the fetal SCN in response to rhythmic maternal signals is not yet known, nor have such rhythms been identified. Therefore, the main goals of our study were (1) to decipher whether the fetal SCN exhibit rhythmicity in cellular processes at developmental stage before the canonical clock develops; and (2) if such rhythmicity exists, what are the rhythmic processes and can they be sorted according to whether they require the presence of functional maternal SCN or respond to systemic rhythms in its absence? The current study pioneers the identification of fetal SCN responses to distinct maternal rhythmic signals through analyzes of transcriptomic and proteomic data in fetal SCN samples collected around the clock at a developmental stage when cell population rhythms in clock gene expression have not yet developed. Recently published biostatistics tools dryR [33] and CompareRhythms [34] allowed us to specifically identify significant rhythms (oscillatory expression patterns) and their origin using a model selection approach for differential rhythmicity analysis (in addition to previously established tools such as MetaCycle and BioCycle) for data acquired from individual fetal SCN samples of different maternal origin. The results revealed a small fraction of functionally related genes and proteins in the rat fetal SCN at E18/19 meeting the criteria for either synchronized, differential, or unique rhythmicity between the analyzed conditions. Most of the observed rhythms were attributed to be directly derived from the maternal behavior/feeding activity (driven either by functional maternal SCN, or as a result of the imposed behavior/feeding rhythm to SCN-lesioned mothers). Additionally, we also identified minor subsets of genes in the fetal SCN whose unique (or differential) rhythmicity could be attributed to signals from the maternal SCN or to the rhythmic maternal signals not dependent on presence of the maternal SCN, respectively.

## Results

### Group description

**Fig 1:**
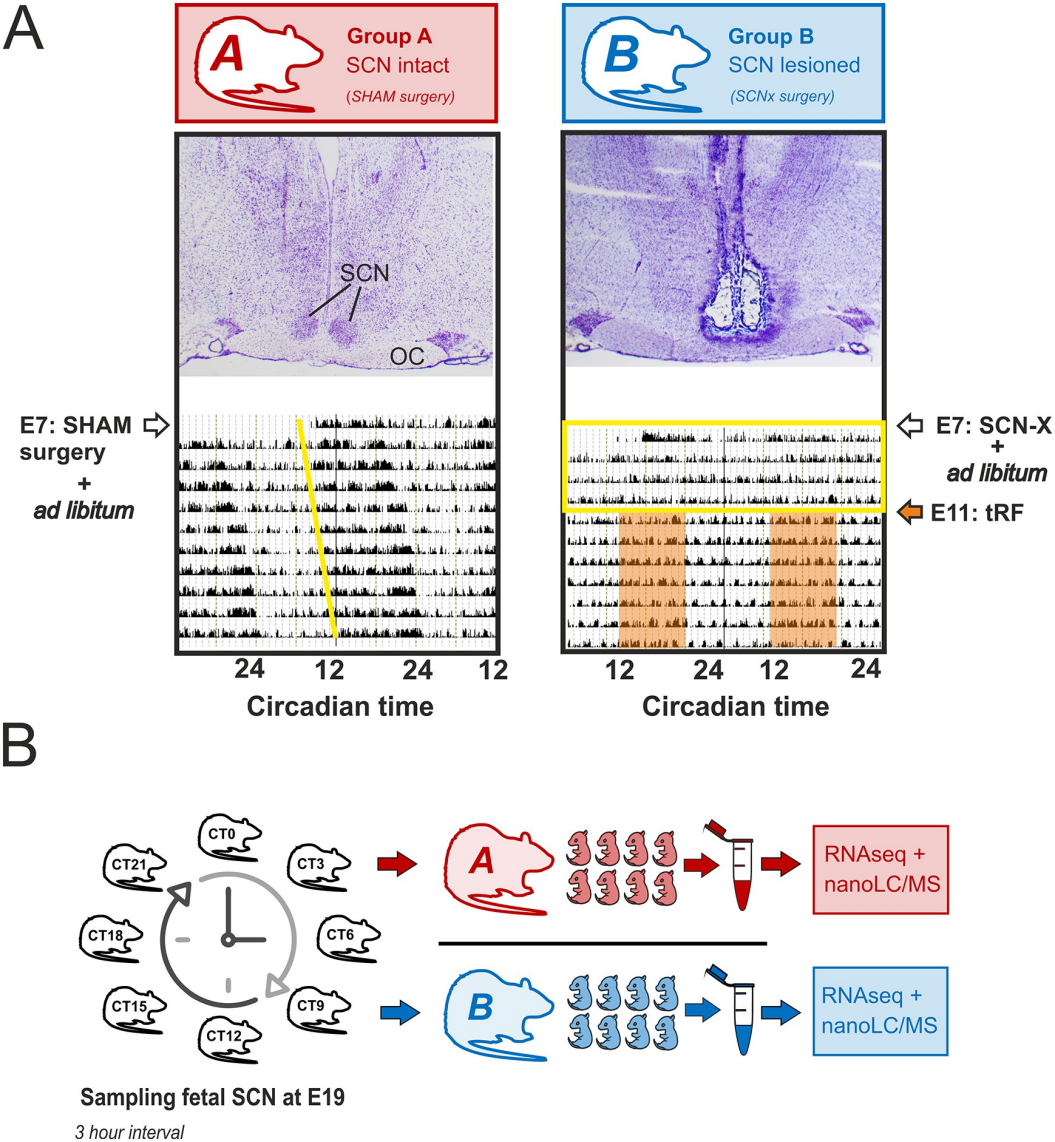
Schematic illustration of experimental design. A total of 32 pregnant rats were divided into 2 groups on the seventh gestational day (E7)—16 rats were exposed to the sham surgery (Group A; transcriptomic study: *n* = 8, proteomic study: *n* = 8) and 16 rats to the surgical procedure for the SCN ablation (Group B; transcriptomic study: *n* = 8, proteomic study: *n* = 8); both groups were maintained in constant darkness. **(A)** Representative histology of the brain sections through hypothalamic region containing the SCN of 1 sham-lesioned (SHAM; Group A) and 1 SCN-lesioned (SCN-X; Group B) pregnant rat and their locomotor activity (double-plotted actograms) recorded in constant darkness (DD) starting on the day of the surgery on embryonic day (E) 7 are shown (OC). Group A was fed ad libitum, while Group B had access to food for only 8 hours per day (tRF) starting from E11 (orange area) to impose a synchronous feeding rhythm on the SCN-lesioned arrhythmic animals. Yellow line in actogram of Group A shows the beginning of free-running locomotor activity in DD; the yellow rectangle in actogram of Group B depicts the interval between E7–E11 when the rats were arrhythmic due to the SCN lesion (used as an evidence for successful operation together with histology). **(B)** Rats were killed around the clock at E19; fetal SCN were collected and analyzed by transcriptomics and proteomics (for more details, see Materials and methods). OC, optical chiasm; SCN, suprachiasmatic nuclei; tRF, time-restricted feeding.

We used fetal SCN from 2 groups of rats maintained in constant darkness (DD) (Fig 1) for RNA-Seq and proteomic analyzes. The control group had sham surgery and was fed ad libitum (Group A). In this group, signals to the fetuses were derived from maternal SCN clock and its complex downstream pathways, including those responsible for timing the feeding and locomotor activity. The SCN-lesioned group was behaviorally arrhythmic in DD and had access to food restricted to 8 hours per day (time-restricted feeding; tRF) to impose a synchronous feeding rhythm (Group B; the activity records are shown in S1 Fig). The 8-hour presence of food did not induce food anticipatory activity that could be distinguished from fragmented bouts of activity detected by infrared detectors in constant darkness. In Group B, direct signals from maternal SCN clock to the fetuses were lacking, with rhythmic signals related to feeding behavior being preserved (provided by tRF) in a similar phase as in Group A (for more details, see Materials and methods).

**Fig 2:**
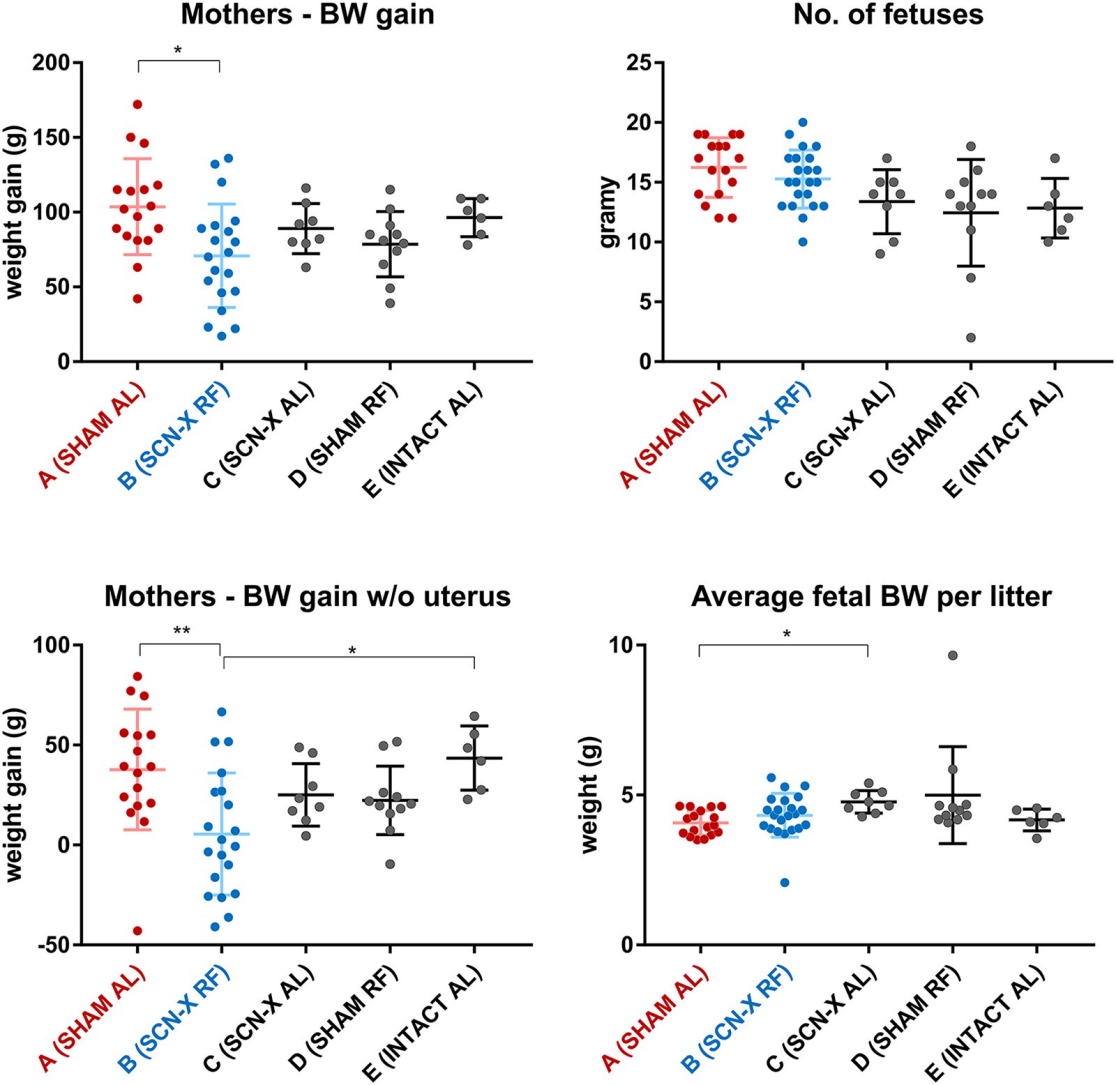
Effects of the SCN lesion and feeding regime on the BW and the size of the litter. The pregnant rats were either fed AL or exposed to tRF regime. Five groups of female rats were analyzed—sham-operated (SHAM) on AL feeding regime (Group A), SCN-X group on tRF (Group B), SCN-X group on AL (Group C), sham-operated on tRF (Group D), and intact controls on AL (Group E). BW gain during pregnancy was significantly lower in mothers of Group B compared to Group A; however, the number of fetuses per litter was not different between all groups. BW gain of mothers with uterus and fetuses removed was significantly lower for Group B in comparison with Groups A and E, and average BW of fetuses in Group A did not differ to Group B but was slightly lower compared to Group C. Analyzed by Kruskal–Wallis test with Dunn multiple comparisons, * *P* < 0.05, ** *P* < 0.01. For raw data, see S1 Data. AL, ad libitum; BW, body weight; SCN, suprachiasmatic nuclei; tRF, time-restricted feeding.

First, we tested whether and how the experimental protocol (mainly surgery and the tRF regime) affected maternal body weight (a measure of potential metabolic challenge) and the number of fetuses in the litter and their weight (a factor that can plausibly affect the variance between fetuses sampled at each time point) (Fig 2). Specifically, we used 3 additional groups of pregnant rats, which were SCN lesioned and fed ad libitum (Group C; *n* = 8), sham operated and exposed to the tRF regime (Group D; *n* = 11), and intact without any intervention (Group E; *n* = 6). The results show that sham surgery of Group A had no effect on the maternal body weight gain and importantly did not decrease number of fetuses in the litter. The rats of Group B had lower body weight gain confirming a higher metabolic challenge (due to exposure to tRF), which they compensated to maintain normal fetal growth. Hence, compared to ad libitum fed animals, food-related and SCN-independent signaling pathways are probably up-regulated in this group. The results showed that the fetal weights and their number in the litters were not different between the experimental groups A and B, in which the transcriptomic and proteomic analyses were performed (the groups C, D, and E were not employed further in these analyses). We also confirmed that the sham operation had no effect on diurnal variation of daily food intake in pregnant rats fed ad libitum (Group A), as they foraged mainly during night hours (approximately 75% of daily food intake; S2 Fig), which was similar to our previous measurements in nonpregnant Wistar rats (36). In Group B, monitoring of feeding behavior was redundant, as rats could forage only during the 8-hour tRF interval.

### RNA-Seq analysis

Temporal gene expression in the SCN of E19 fetuses was investigated using RNA-Seq (for details on sample collection, see Materials and methods). We obtained 11.9 to 17.9 million reads per sample (S3 Fig), 92.6% to 93.8% of which were uniquely mapped to the rat genome. Exactly 6,973 transcripts from Group A and 6,992 transcripts from Group B had average fragments per kilobase of transcript per million mapped reads (FPKM) values > 10 across all time points, while approximately 13,000 transcripts in both groups had average FPKM values > 1. We used deseq2 to analyze all transcripts that had at least 1.5 raw count per million mapped reads in at least 3 sample libraries and were assigned a unique gene ID (*n* = 12,844). There was no difference in overall expression levels between the 2 groups, as none of the transcripts showed significant up- or down-regulation (lowest multiplicity adjusted *P* = 0.055). This was reflected in the incomplete separation of both groups by whole-transcriptome clustering analysis and likely reflects time-dependent in-group differences.

**Fig 3:**
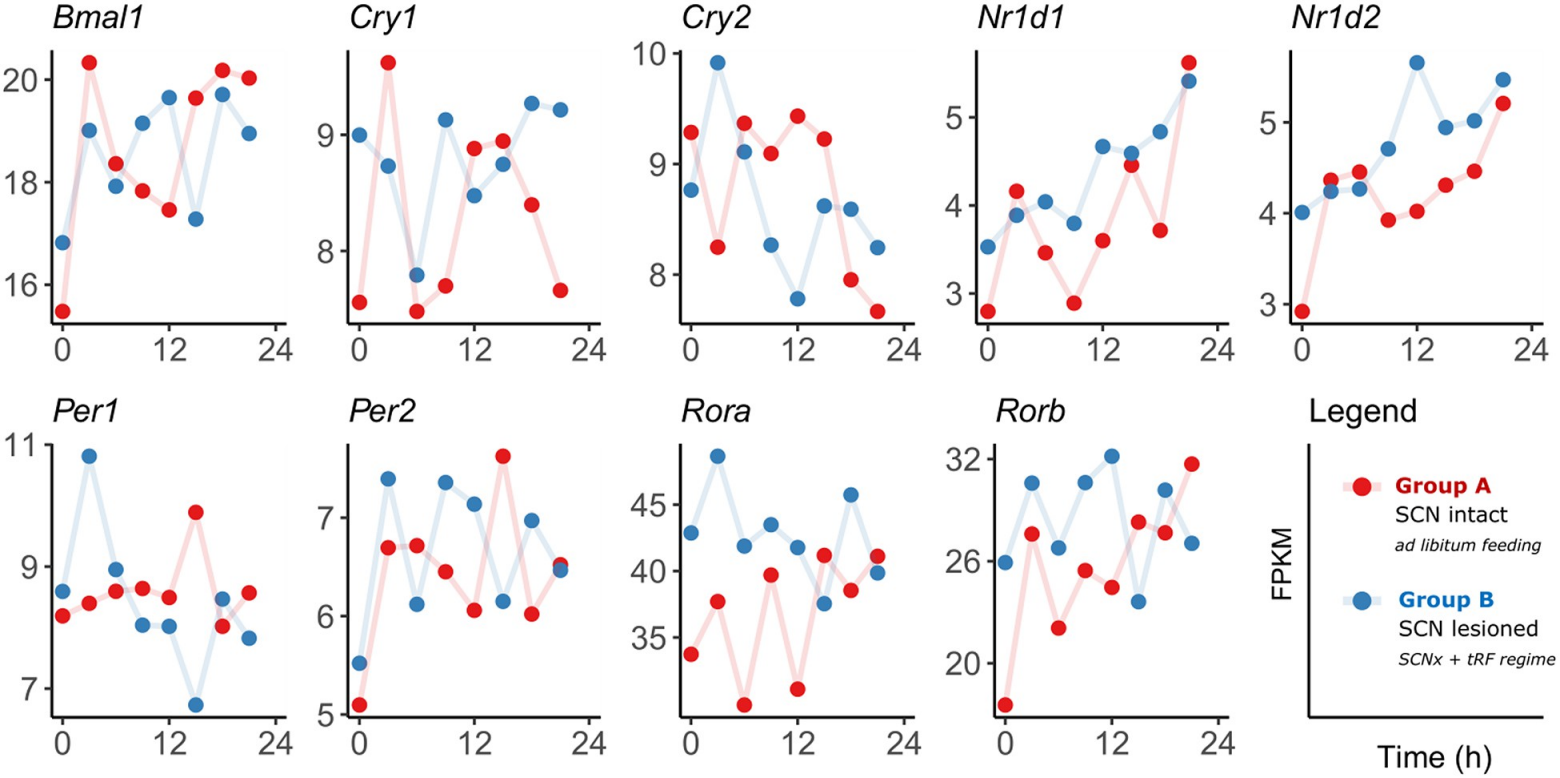
Temporal expression pattern of canonical clock in the E19 fetal rat SCN in Group A (SHAM, ad libitum, red) and Group B (SCN-X, tRF, blue). For raw data, see NCBI’s GEO GSE183172. SCN, suprachiasmatic nuclei.

All major clock genes were detected at low levels with average FPKM values < 10 (Fig 3), except more highly expressed *Bmal1* (FPKM approximately 20) and *Rora/b* (FPKM approximately 20 to 40). Various rhythm analysis tools were used to identify the presence or absence of rhythmicity, including conventional approaches (MetaCycle, eJTK), model selection methods for differential rhythmicity comparison (dryR, CompareRhythms), and machine learning tool BioCycle, with thresholds for multiple testing (FDR < 0.05) and a Bayes information criterion weight (BICW > 0.5) for model selection methods applied. Transcripts of none of the major clock genes were significantly rhythmic as confirmed by all tools (S2 Data). There was an exception for *Per1* expression selectively in Group B (BICW = 0.73 dryR, BICW = 0.81 CompareRhythms).

**Fig 4:**
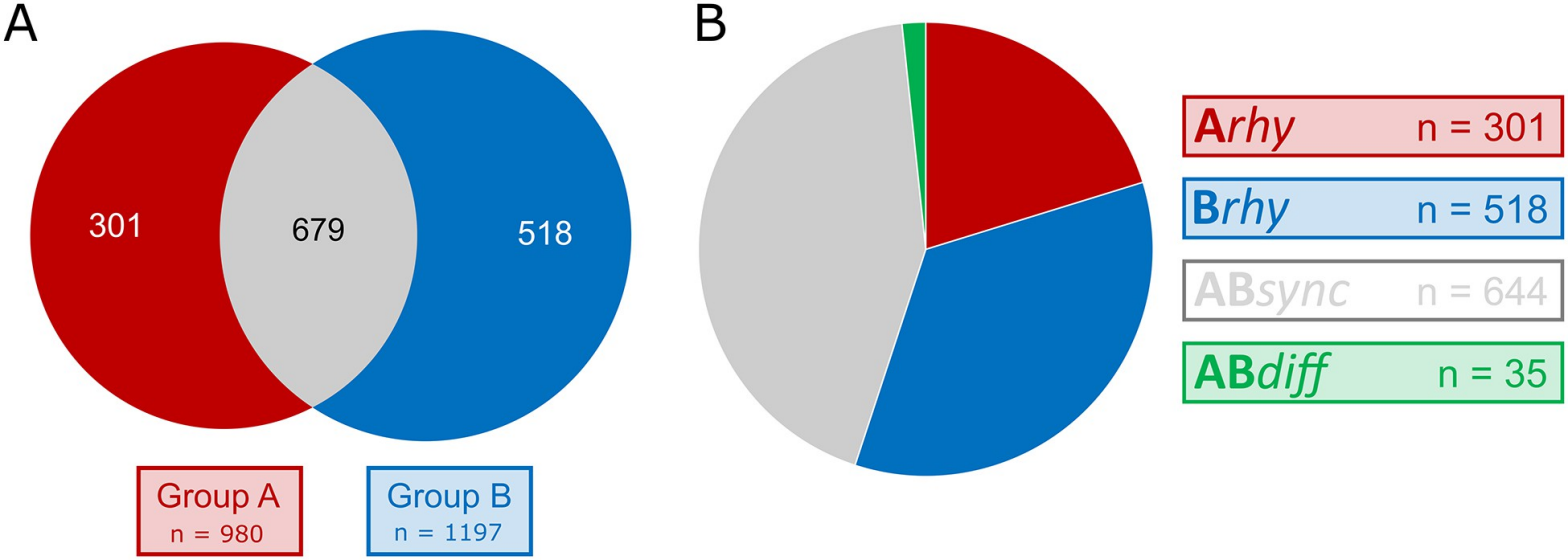
Distribution of rhythmically expressed genes in the E19 fetal rat SCN. **(A)** Transcripts identified as rhythmic by dryR analysis in either Group A (red), Group B (blue), or both groups (gray). **(B)** Transcripts assigned to their respective subsets: rhythmic in Group A (*Arhy*, red), rhythmic in Group B (*Brhy*, blue), and transcripts rhythmic in both groups with similar (*ABsync*, gray) or different circadian parameters (*ABdiff*, green). SCN, suprachiasmatic nuclei.

Model selection approaches were used to compare rhythms between Group A (SHAM, ad libitum) and B (SCN-X, tRF) and assign transcripts to subsets according to the presence or absence of rhythmicity in their expression (*n* = 12,844) with application of an additional BICW > 0.5 (*n* = 9,174 for dryR; *n* = 8,167 for CompareRhythms). The majority of transcripts were found to be nonrhythmic in both groups (*n* = 7,676 for dryR; *n* = 5,346 for CompareRhythms). Because the rhythms in the expression of clock genes are necessary for the operation of an endogenous fetal clock mechanism and they were not detectable by any of the previously mentioned methods, we assume the rhythmic genes in the remaining subsets identified by dryR (*n* = 1,498; Fig 4) and CompareRhythms (*n* = 3,828; S3 Data) were of maternal origin. They included transcripts with similar rhythmic parameters (phase and amplitude) in both groups (*ABsync*), differentially rhythmic genes (*ABdiff*), transcripts rhythmic only in Group A (*Arhy*), and those rhythmic only in Group B (*Brhy*).

**Fig 5:**
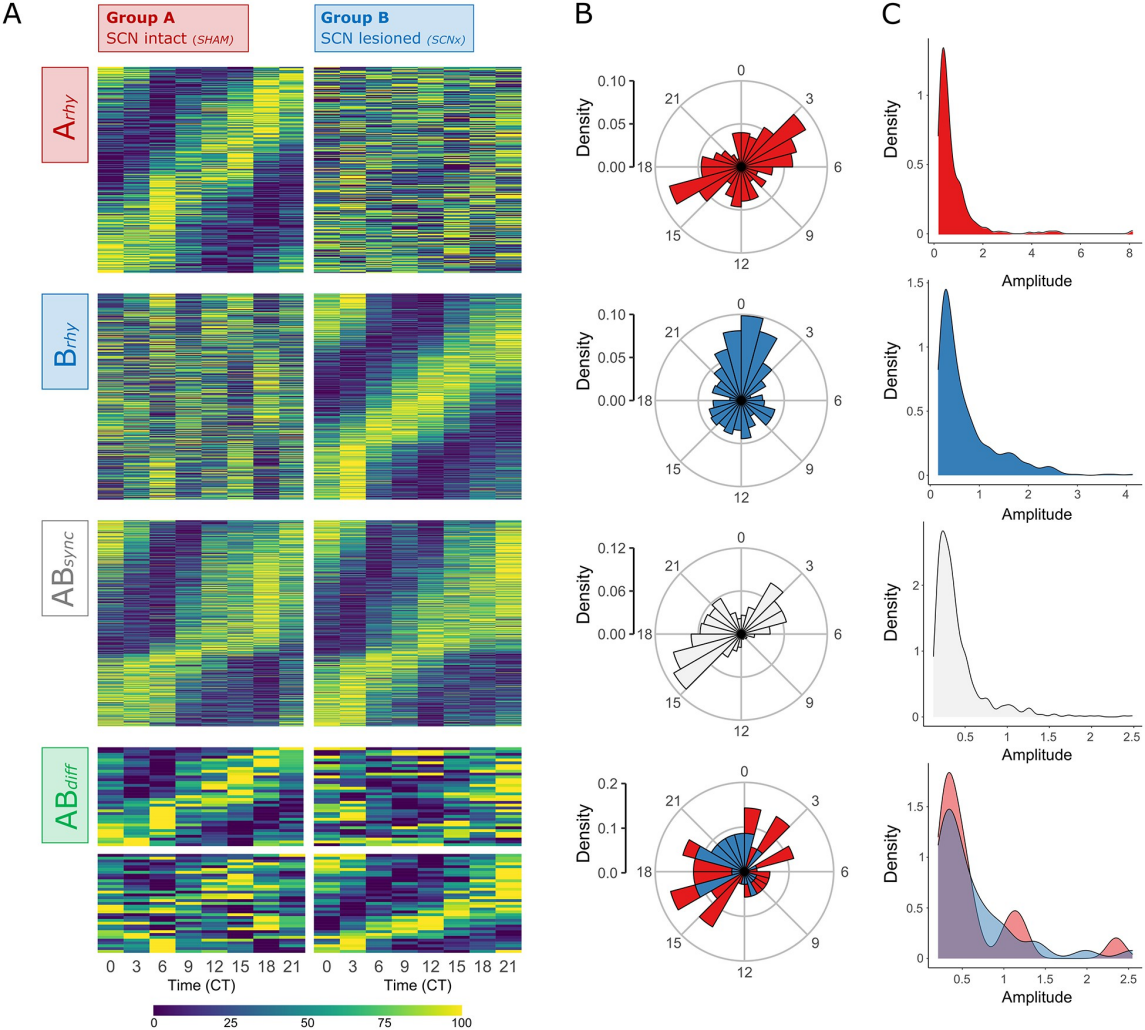
Circadian parameters of rhythmically expressed genes in the E19 fetal rat SCN. **(A)** Heatmaps of gene subsets identified by dryR depending on presence/absence of rhythmicity in either or both experimental groups. Transcripts classified as nonrhythmic in both groups and unclassified transcripts (BICW < 0.5) are not visualized (see S3 Data). Transcripts rhythmic in Group A (left), but not in Group B (right), or vice versa, were assigned to Model *Arhy* (*n* = 301) and *Brhy* (*n* = 518), respectively. *ABsync* (*n* = 644) includes transcripts rhythmic in both groups with similar phase and/or amplitude, and *ABdiff* (*n* = 35) with different phase and/or amplitude, respectively. Heatmaps of normalized rhythmic mRNA levels in the fetal SCN at 3-hour interval for Groups A (left) and B (right). **(B)** Phase and **(C)** amplitude distribution of transcripts classified as rhythmic within the respective dryR models (for *ABdiff*–Group A: red, Group B: blue). For raw data, see NCBI’s GEO no. GSE183172 and S3 Data. BICW, Bayes information criterion weight; SCN, suprachiasmatic nuclei.

Most transcripts were rhythmic in Groups A and B, according to both dryR (approximately 43%) and CompareRhythms (approximately 25%), and the majority of these rhythmic genes reached their peak in expression between CT15 and CT21, with a smaller group of genes in the opposite phase (peaking at CT3-6, respectively). The dryR analysis (Fig 5) identified 644 transcripts of similar circadian parameters (ABsync) and differential rhythmicity in only 35 (approximately 2%) genes (ABdiff), while CompareRhythms found 976 synchronous (Absync) and 294 (approximately 8%) differentially rhythmic genes (ABdiff). Transcripts rhythmic only in Group B (Brhy; *n* = 518 dryR; *n* = 902 for CompareRhythms) made up approximately 35% (approximately 24% for CompareRhythms) mostly peaking around CT21-3. Those rhythmic only in Group A (Arhy; *n* = 301 for dryR; *n* = 458 for CompareRhythms) made up approximately 20% (approximately 12% for CompareRhythms), with the majority of associated genes peaking between CT15-18 (and to less extent CT3-6, in similar distribution to the ABsync subset).

**Fig 6:**
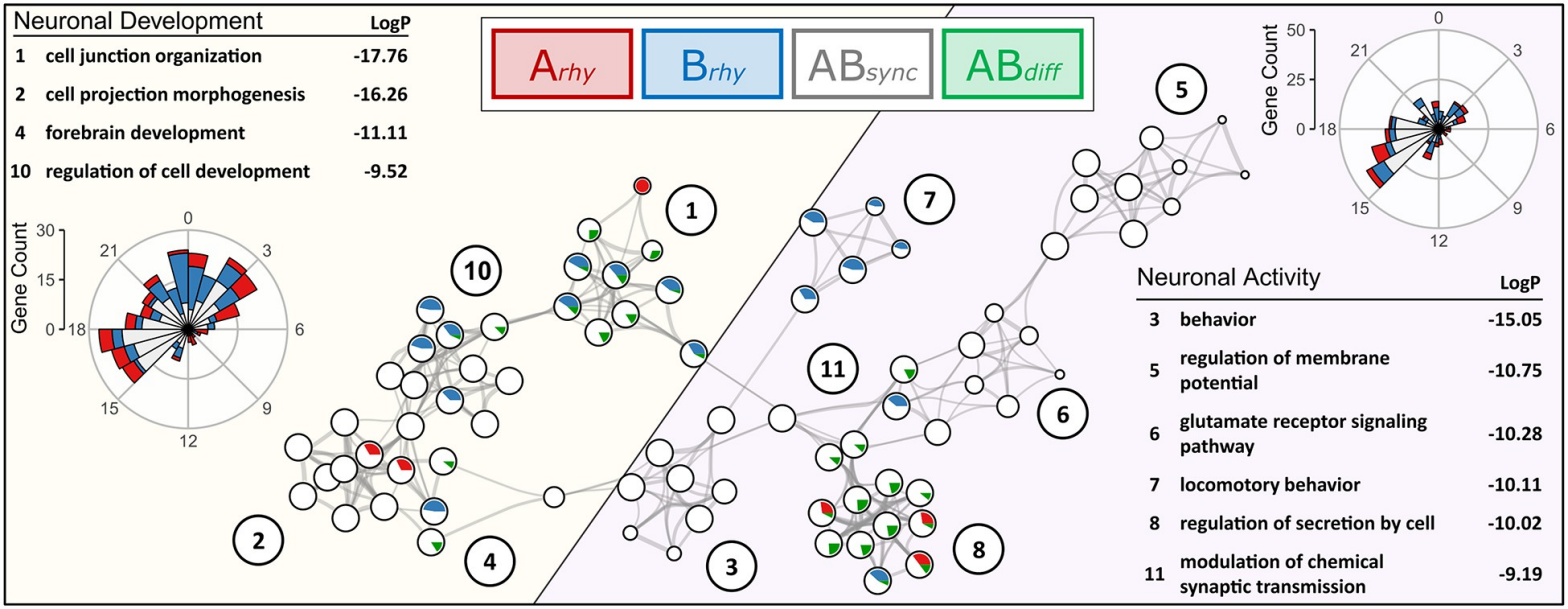
GO Biological Process enrichment network of subsets identified by dryR. Statistically significant enriched GO Biological Process terms were clustered based on similarities among rhythmic genes found within the subsets identified by dryR. The term with the lowest *P* value within each cluster was chosen as a representative label. Each GO term is represented by a circle node, with size proportional to the number of genes. Color and size of the pie sectors represent the number of hits originated from each respective subset (transcripts rhythmic in Group A—*Arhy* = red, transcripts rhythmic in Group B—*Brhy* = blue, transcripts rhythmic in both groups with similar phase—*ABsync* = white, transcripts rhythmic in both groups with different phase—*ABdiff* = green). The network represents 10 of the 12 most significant clusters, with blood vessel development (9) and sensory organ development (12) omitted from the figure because they were not connected to other clusters within the network. Two main branches are highlighted: The right branch (purple) is associated with neuronal activity, while the left branch (yellow) is comprised of genes associated with neuronal development. -LogP-values of the enrichment and phase distribution of genes found within the respective branches are shown on the far right (neuronal activity) and left (neuronal development), respectively. For raw data, see S4 Data. GO, Gene Ontology.

For enrichment analysis of these subsets of rhythmically expressed genes, we used both single- and multi-gene list enrichment analysis on MetaScape targeting GO Biological Process, Molecular Function and Cellular Component, as well as KEGG and Reactome pathways to assign functions to gene subsets representing the models identified by dryR and CompareRhythms. The most notable observations point toward an enrichment of genes associated with neurodevelopment, (synaptic) signaling and activity (S4 Data). The relationship of these subsets (based on multi-gene list GO-BP enrichment analysis of the dryR subsets) is shown in a network visualized with Cytoscape, representing 10 of the 12 most highly significant clusters assigned by MetaScape (-log(p) > 9), as clusters 8 and 12 were not connected to any of the other nodes via edges and were therefore addressed separately. The most significantly enriched GO-BP term from each cluster was used as term description. Two main branches can be observed in the network (Fig 6, highlighted in yellow and purple, respectively), in which color and size of the nodes represent the number of genes originated from each respective subset: *Arhy*, *Brhy*, *ABsync*, or *ABdiff* (highlighted in red, blue, white, and green, respectively).

The right branch (purple) is associated with neuronal activity and is comprised of the clusters *behavior (3*, -log(p) = 15.05*)*, *regulation of membrane potential (5*, -log(p) = 10.75*)*, *glutamate receptor signaling (6*, -log(p) = 10.28*)*, *locomotory behavior (7*, -log(p) = 10.11*)*, *regulation of secretion by cell (8*, -log(p) = 10.02*)*, and *modulation of chemical synaptic transmission (9*, -log(p) = 9.19*)*. Most of the genes (*n* = 318) are rhythmic in both groups with similar phase and amplitude (*ABsync* approximately 56%), followed by genes rhythmic only in Group B (*Brhy* approximately 27%), only in Group A (*Arhy* approximately 14%) and differentially rhythmic genes (*ABdiff* approximately 4%). Numerous genes within this branch are associated with catalytic and transporter activity or encode for transmembrane signal receptors and downstream signaling enzymes. This includes various relevant subunits of adenylate cyclases, ionotropic and metabotropic glutamate/GABA receptors, sodium/potassium-transporting ATPases, Ca-, K-, Na-, and Cl-channels. The majority of rhythmically expressed genes associated with neuronal activity peaked between CT15-18 (Fig 6, S3 Data).

The left branch (yellow) represents genes associated with neurodevelopment and is made up of the clusters *cell junction organization (1*, -log(p) = 17.76*)*, *cell projection morphogenesis (2*, -log(p) = 16.26*)*, *forebrain development (4*, -log(p) = 11.11*)*, and *regulation of cell development (10*, -log(p) = 9.53*)*. As in the right branch, most of the genes (*n* = 335) were mutually rhythmic in both groups with similar phase and amplitude (*ABsync* approximately 52%), followed by genes rhythmic only in Group B (*Brhy* approximately 28%), only in Group A (*Arhy* approximately 16%) and differentially rhythmic genes (*ABdiff* approximately 3%). Additional genes that are found in multiple clusters of this branch include signaling enzymes, growth factors, and receptors such as *Egfr*, *Akt1*, *Map2k1*, *Notch1*, *Stat3*, *Lhx2/9*, *Ncor1/2*, and *Plcb1* (*ABsync*), as well as *Pdgfra*, *Tgfb1*, and *Vegfa* (*Brhy*) or *Ntrk2/3* (*ABdiff*). Rhythmically expressed genes associated with neuronal development peaked between CT15-3 (Fig 6, S3 Data).

We examined the expression of key receptors binding molecules previously associated with maternal entrainment of the fetal circadian system, namely hormones melatonin [35,36], glucocorticoids [21], dopamine [37], and the SCN major neurotransmitters vasopressin and vasoactive intestinal polypeptide [38]. The level of *Mtnr1a*, a gene encoding melatonin receptor 1 (MT1), was very low (FPKM = 0.6) and *Mtnr1b* (MT2) was undetectable. Glucocorticoid receptor (*Nr3c1*), Vasopressin (*Avp*), and its receptors (*Avpr1a* and *Avpr2*) were expressed at higher levels (FPKM = 1.5–6). Vasoactive intestinal polypeptide (*Vip*) and its receptor (*Vipr2*), as well as Dopamine receptor 1 (*Drd1*) exhibited the highest expression (FPKM = 10–15) of the investigated transcripts, while Dopamine receptor 2 (*Drd2*, FPKM = 0.4) was detected at expression below threshold. All mentioned genes were identified as nonrhythmic in both Groups A and B by dryR, with the exception of *Drd1* (BICW = 0.51) observed as rhythmic in both groups. *Avpr2* was initially also identified as rhythmic in Group A (BICW = 0.35 in dryR) but did not pass the confidence threshold and was therefore left unassigned (S3 Data). Most of the rhythmically expressed genes were assigned to be rhythmic in both Groups A and B (*ABsync*), with all previously mentioned GO term clusters significantly enriched in the respective *dryR* subset (Fig 6, white). Additionally, GO term *rhythmic process (GO*:*0048511*, *-log(p) = 6*.*26)* was found to be significantly enriched within the *ABsync* subset due to the presence of genes such as *Adcy1*, *Adora1*, *Drd1*, *Gabrb1*, *Grin1/2b*, *Hspa8*, *Ncor1/2*, *Sox14*, or *Tp53* within this subset. These (and other) genes could potentially provide a link between observed oscillations present in response to rhythmic maternal signals and the establishment of independent rhythms in the offspring during later development.

The subset of genes found rhythmic in Group A (*Arhy*) was represented twice in the network (Fig 4, red) due to the presence of genes such as *Avp*, *Hdac5/9*, *Hspa4*, *Pax6*, *Shh*, and *Tbx2/3*, as well as further subunits of previously mentioned receptor/transporter genes (e.g., *Cacna1g/i*, *Grik1*). Individual analysis of the subset revealed an exclusive enrichment for the GO term *cellular response to leucine* (GO:0071233, -log(p) = 4.89), including genes for sestrins *Sesn1/2/3*, *Mtor*, *Map3k5* (also known as *Ask1*), *Gck*, and *Slc38a2*.

The dryR model comprised of genes found rhythmic in fetal SCN of Group B litters (*Brhy*) displayed enrichment in 5 of the 12 most significant clusters (Fig 4, blue), including the clusters *blood vessel development* (9) and *sensory organ development* (12), which are not connected to other top 12 clusters and not visualized in the network (S4 Data). Individual analysis also revealed enrichment in transmembrane receptor protein tyrosine kinase signaling pathway (GO:0007169). These clusters contain a variety of growth factors, receptors, and signaling enzymes rhythmic only in Group B, such as *Bmp7*, *Csf1* and *Csf1r*, *Dlx1/2*, *Egfl*, *Fgf1* and *Fgfbp3*, *Igf2r* and *Igfbp2/4*, *Pdgfra*, *Prkcd* and *Prkcq*, *Tgfb1* or *Vegfa*.

dryR identified only 35 differentially rhythmic genes (*ABdiff*), but 4 of the 12 GO terms were significantly enriched in this subset (Fig 4, green). Member genes of these clusters include *Gria3/2a*, *Slc17a6* and *Slc32a1*, *Hspa5*, *Ntrk2/3*, *Stxbp5*, *Syt5* and *Cacna1d*. The differential phases are shown in S3 Data.

### Proteomics analysis

**Fig 7:**
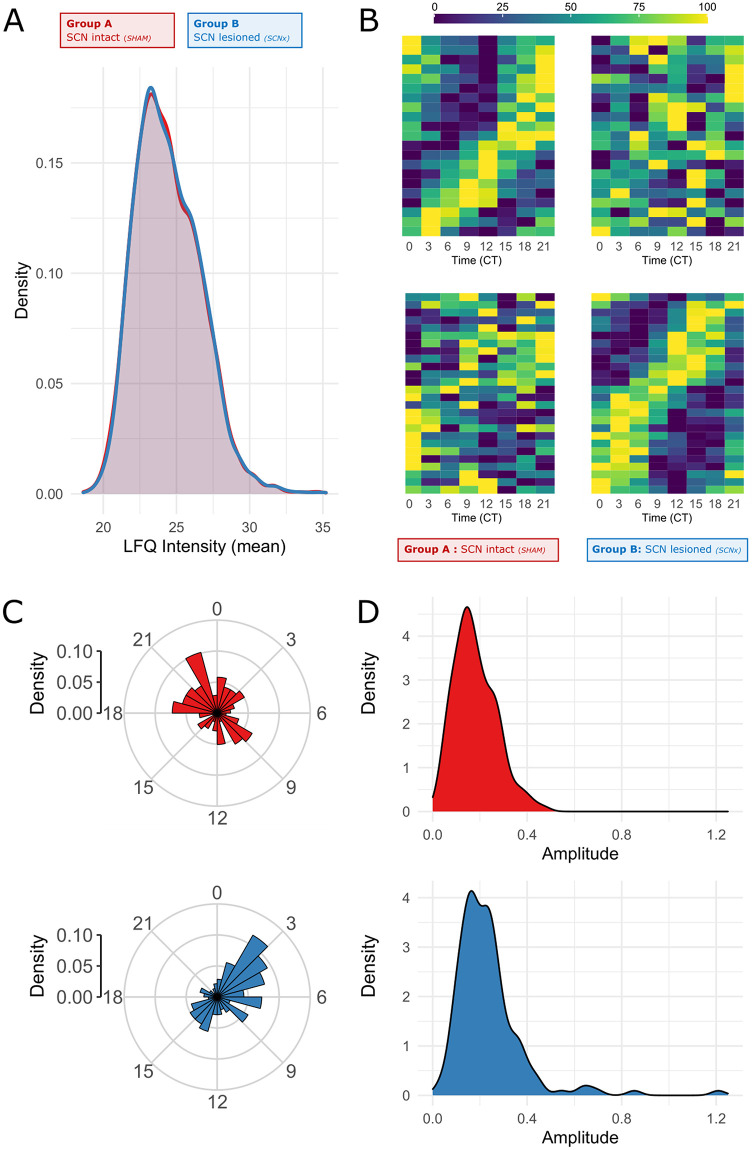
Proteomics. LFQ intensity values of 3,707 unique proteins detected in Groups A (SHAM, ad libitum) and B (SCN-X, tRF) across 8 time points (CT0-CT21). **(A)** Density plot of mean LFQ values shows similar distribution in both groups (*t* test, *P* = 0.69) **(B)** Heatmaps of normalized protein levels in the fetal SCN for Groups A (left) and B (right) characterized as rhythmic via BioCycle (*p* < 0.05 in Group A [top] or Group B [bottom]). **(C)** Phase and **(D)** amplitude distribution of proteins rhythmic in Group A (red) and Group B (blue). For raw data, see S5 Data. LFQ, label-free quantification; SCN, suprachiasmatic nuclei.

We used independent batch of identically sampled fetal SCN from mothers of Groups A and B for liquid chromatography coupled with mass spectrometry to identify label-free quantification (LFQ) values of 3,707 unique proteins. The average LFQ values ranged from 18.9 to 35.2 with similar distribution in both groups (*t* test, *P* = 0.69; Fig 7A). The major canonical clock proteins were undetectable at E19, with the exception of casein kinases CSNK1D (LFQ 25.1) and CSNK1E (LFQ = 22.1).

Normalized LFQ values were analyzed by various conventional tools for rhythm detection, but no significant circadian changes in protein levels across time points were found by any of the methods and the majority of genes identified to be expressed rhythmically on transcriptomic level were undetectable or not reported in the proteomic analysis.

Using BioCycle as a deep learning system capable of estimating the periodic signal in noisy datasets revealed that both the A and B group datasets were remarkably stable over time, with a maximum of 5% difference between peak and trough values. Nevertheless, BioCycle identified 21 proteins in Group A and 26 proteins in Group B as potentially rhythmic (unadjusted *P* < 0.01; Fig 7B–7D).

Additionally, normalized LC-MS data of proteins identified as rhythmic on transcript level (*n* < 500) were compared via CompareRhythms, in spite of apparent limitations of this approach. Only 20 genes (= 4%) were identified as rhythmic in both transcriptomic and proteomic analyses via model-selecting methods (with 9 displaying BICW < 0.5). Most of those genes/proteins were assigned to *ABsync* (*n* = 11), followed by *Brhy* (*n* = 5) and *Arhy* (*n* = 4) by both *dryR* and CompareRhythms. Only *Cdk3* was assigned to different subsets by the 2 methods, with *Brhy* according to dryR (BICW = 0.39), while CompareRhythms identified it as *ABdiff* both on transcript and protein level (BICW = 0.58 and 0.60, respectively).

Results of rhythm detection analyses are summarized in S5 Data. Due to the small number of rhythmic proteins identified both by deep learning system BioCycle and model selection tool CompareRhythms, we did not employ any additional enrichment analyses.

## Discussion

We used 2 recently published model-selecting methods (dryR [33] and CompareRhythms [34]) for analysis of circadian transcriptomic data in the rat fetal SCN samples collected at developmental stage (E18 to E19) in 3-hour intervals around the clock, in order to avoid overestimation of the number of differentially rhythmic genes (compared to common hypothesis-testing approaches). We designed the experimental protocol so that the fetuses were exposed to rhythmic signals derived from mothers, which had either intact SCN (Group A) or had the SCN surgically ablated and their behavior/feeding rhythm was imposed by tRF (Group B). The applied modeling approaches allowed us to differentiate between genes/proteins in the fetal SCN, which responded to either of the experimental conditions. We confirmed that circadian rhythmicity could not be detected for expression profiles of the canonical clock genes (with the exception *Per1* in Group B) in the fetal SCN samples using a variety of rhythm detection methods. Additionally, analysis of proteomic data revealed that levels of canonical clock proteins were below detection limit, in line with the previous report on absence of their immunopositivity in the rat E19 SCN [13]. Because our data confirmed that the intrinsic rhythmicity in the population of SCN cells had not yet developed at that fetal stage, we can assume that all rhythms detected in our transcriptomic study by the model selection methods dryR and CompareRhythms represent a response to signals of maternal origin. However, we cannot formally exclude the possibility that a subset of these rhythms is generated by rhythmic fetal processes that are distinct from the canonical molecular clock.

Based on the structural development of the SCN (as summarized in the Introduction), we expected that rhythmic responses to external signals in the fetal SCN may represent only a distinct subset of cellular processes, and if present, they would be of low amplitude. This was indeed confirmed by analyzes of the transcriptomic and proteomic datasets. We are aware that the observed shallow rhythmicity raises the question of a degree of reliability of rhythm detection, especially for the samples that had to be pooled for technical reasons. However, the pooling of fetal SCN samples is unlikely to play a role in identifying circadian rhythms resulting from maternal signals in otherwise arrhythmic, immature fetal SCN tissue lacking a functional circadian system, since in this experimental design, the same maternal signals interact with the SCN of all fetuses in the litter in synchrony, and therefore, they should trigger litter-level responses. We have attempted to minimize the amount of false negatives by controlling the quality of the samples at multiple steps. To avoid false positivity in the identification of rhythmic profiles, we increased the stringency for the model selection criteria above default confidence thresholds (see methods for details). Additionally, at least 3 lines of evidence support the validity of the analyzed outputs:

1. *Consistency of data with previous studies*. As mentioned, none of the clock genes were identified as significantly rhythmic (FDR < 0.05) in the control group A by BioCycle, eJTK, and MetaCycle analyses. This is consistent with data previously shown for RT-qPCR–analyzed individual SCN samples collected at the same gestational stage in the same rat strain [13,14] and other species [15,16].
2. *GO term enrichment*. The observed functional clusters within the subsets identified by dryR provide strong support for the validity of the experimental approach. If the identification of rhythmic profiles were to result from random variation in expression levels at individual time points, the identified rhythms would likely lack obvious functional connectivity and highly significant enrichment within the large datasets. Additionally, to avoid the false positivity and identify top hits, we applied stringent thresholds for expression level and rhythmicity. Furthermore, even though the subsets identified by CompareRhythms were larger than the dryR subsets, MetaScape analyses still reported similar enrichment patterns of almost identical gene hits within the enriched clusters.
3. *Substantial overlap between both groups*. The fact that the largest subsets of genes were identified as rhythmic in both groups by both model-selecting methods lends more weight to the validity of the data, as it is reasonable to expect a large proportion of rhythmically responding pathways in both groups being shared and (mostly) synchronized. This assumption is based on the experimental design, in which the activity/rest windows in the ad libitum fed mothers with intact SCN (Group A) corresponded to the feeding/fasting windows in the SCN-lesioned mothers (Group B). Indeed, only a negligible amount of the rhythmically expressed genes differed in phasing in dryR analysis. Although CompareRhythms identified more genes as differentially rhythmic, the reported changes in rhythm parameters between the 2 groups were comparatively minor, especially in light of the generally shallow rhythmicity at this developmental stage, and are most likely a result of the higher metabolic pressure imposed on the mothers in Group B due to the tRF regime, which could potentially affect expression of some genes. Hence, we also confirmed usability of model selection methods like dryR and CompareRhythms for analysis of shallow rhythmicity in the fetal SCN (with minor limitations).

We also analyzed the impact of our experimental procedure on the fetal weights and number of fetuses in the litters, which could have potentially affected the outcome of the study. We found that exposure of mothers to tRF had no effect on the number of fetuses in the litters and their weights at E19 (in spite of the tRF caused lower body weight gain in the mothers, compared to the ad libitum fed groups). This result was in accordance with our previous study in which pregnant rats were maintained on similar tRF regime and constant light [28]. The results suggest that in our experimental protocol, tRF-induced metabolic pressure inflicted on rats during pregnancy may be compensated by reduced gain in the mothers’ own body weight in order to protect fetuses from potential malnutrition. Altogether, based on all the above summarized predictions, we believe that the experimental protocol and the biostatistics approaches allow us to reliably confirm the presence or absence of gene expression rhythms in the fetal SCN samples.

We hypothesized that maternal signals may drive early rhythmical processes involved in cell-to-cell signaling and neurodevelopment in the fetal SCN. This prediction was confirmed by identifying 2 distinct branches of enriched GO term clusters. One branch was dominantly comprised of genes involved in neuronal activity and coding for signaling molecules and pathways (such as a variety of signaling enzymes, transporters, receptors, voltage/ion-gated channels, major neurotransmitters such as glutamate and GABA, etc.), and the other branch was comprised of genes involved in neuronal development (such as neuron projection pathways, morphogenesis, cellular organization and differentiation, etc.). Expression of most of the genes in the neuronal activity branch peaked during the activity/feeding phase of one or both groups of mothers, suggesting that maternal activity/feeding state may trigger neuronal activity in the fetal SCN resulting in daily variation of related gene expression. The neurodevelopment branch exhibits a similar pattern, with expression peaks spanning over the activity and the beginning of inactivity state. This branch includes various genes associated with growth factor signaling, many of them uniquely rhythmic in Group B, which thus could be more affected by the metabolic effects of the tRF regime on the mother, in comparison to genes within the branch associated with neuronal activity (like GABA- or glutamatergic signaling).

Additionally, the model-selecting methods allowed us to distinguish between subsets of genes responding to rhythmic maternal signals of different origin, that is, those derived from direct maternal SCN signals (*Arhy*) or from other maternal signals (*Brhy*), as well as genes responding to both mechanisms, either in synchrony or out of phase (*ABsync* and *ABdiff*, respectively; S3 Data). Genes rhythmically responding to maternal signals irrespective of their origin (*ABsync*) represented the largest subset identified by dryR. This reflects the fact that the maternal SCN clock can potentially use multiple output pathways for synchronization of the periphery, and the behavior/feeding rhythm contributes as one of the most powerful entraining signals even for the fetal SCN [39–41]. Slightly less abundant subset contained genes rhythmic under condition of maternal signals enhanced by temporal restriction of the food availability and omitting signals directly driven by the maternal SCN (*Brhy*). This suggests that the conditions can directly provide the fetal SCN with a burst of signals that induce rhythmic responses in expression of a significant subset of genes in the fetal SCN. Interestingly, less genes were rhythmic in presence of signals directly driven by maternal SCN, which may include the SCN-driven humoral signals oscillating in the maternal body and, compared to rRF, relatively weaker ad libitum feeding-related signal (*Arhy*). Finally, the set of genes expressed rhythmically but with different phases in both groups was the least represented (*ABdiff*) in the results of the dryR analysis. This is expectable because fetuses of both groups were collected according to maternal internal time as determined based on the beginning of the SCN-driven or food-imposed activity. Noteworthy, the result provides a good confirmation that this determination of “endogenous time” based on the food-induced bout of activity in the arrhythmic SCN-lesioned mother was relevant. The very small subset of genes expressed out of synchrony was likely not responding solely to maternal activity, but rather by a more complex signaling mechanism. Interestingly, the differentially rhythmic genes that exhibit shifts in phase between the 2 groups appear to align with the peaks of uniquely rhythmic genes of their respective groups (differential peaks of *ABdiff* align with peaks of *Arhy* and *Brhy*, respectively). It is noteworthy that CompareRhythms identified more differential rhythms than dryR. It can be explained by the respective sensitivity toward variations in rhythm parameters of both model selection methods, since genes identified as *ABdiff* by CompareRhythms exhibit a similar general distribution in circadian rhythm parameters as those observed in dryR (while being assigned to *ABsync*). This is also confirmed by the fact that all transcripts identified as differentially rhythmic by dryR are also featured in the (much larger) subset of *ABdiff* in CompareRhythms. Given the aim of our study, we therefore decided to focus our attention on the dryR subsets, in order not to overestimate minor variations in the circadian parameters of (already shallow) rhythms when assigning differential rhythmicity between the 2 experimental groups.

Identification of genes in the fetal SCN that were assigned to be responding to distinct rhythmic signals delivered directly from the maternal SCN, or from behavior/feeding state omitting the direct SCN signals, provides a unique baseline for future exploration of how maternal stimuli contribute to fetal development of this structure. Our data revealed that in the early fetal SCN development, they may substitute for an absent intercellular web of synapses and drive cell population rhythms before the SCN clock fully matures. It remains to be clarified which of the multiple rhythmic processes are responsible for establishment of synchrony between the fetal and maternal clock. Noteworthy, the GO term *rhythmic process* enriched in the gene *ABsync* subset (rhythmic in both Groups A and B) provides a promising list of candidates. We would like to point at the fact that our study was not designed to determine whether the fetal SCN at this developmental stage can exhibit any rhythmicity in the complete absence of rhythmic maternal signals, since addressing this research question will require a currently unavailable technique for in vivo monitoring of rhythms in the SCN of individual fetuses. Such an approach would merit further investigation in the future in order to add an additional layer of observations to complement our results. Additionally, future studies should identify genes responding selectively to maternal feeding signals. Regardless, our major finding that most rhythmic transcripts peaked in the fetal SCN at the time corresponding to maternal behavioral activity/feeding suggests that these early rhythms represent responses to the maternal signals.

The results of the proteomic study were less conclusive. Similar to previous mass spectrometry studies of the adult SCN [42–46], we could not detect any core clock proteins in our fetal samples. However, we also found only a limited number of other rhythmic proteins, which exhibited very low amplitudes. Specifically, we did not identify rhythms in any potentially rhythmically regulated proteins, such as rate limiting transcription and signaling factors or membrane receptors, which might be due to their low abundance relative to the cytoplasmic proteins. Furthermore, less than 500 genes that were identified as either synchronized or differentially rhythmic by model selection methods were detected on the protein level, with only 20 of those proteins also identified as rhythmic based on the normalized LC-MS data. It is possible that applying advanced quantitative phosphoproteomics [47] would provide more information on circadian regulations in the fetal SCN tissue. Alternatively, it is likely that as the SCN matures during fetal development, majority of proteins that are abundant at this stage serve as essential housekeeping proteins, which are generally not subject to rhythmic regulation.

In conclusion, our transcriptomic results provide novel insights into the previously unnoticed scope and diversity of rhythmically expressed genes in the fetal SCN just before cell population rhythms in locally expressed clock genes start to manifest coherently. Although we cannot rule out the possibility that a subset of these rhythms may be generated by processes distinct from the canonical molecular clock, the fact that most rhythmic transcripts in the fetal SCN peak synchronously with maternal signals suggests that these early rhythms represent responses to maternal signals. The unexpected broadness and specificity of responsiveness of the SCN cells to maternal signals stresses the importance of a healthy maternal circadian system during pregnancy and points at the potential impact of the absence of such signals in prematurely delivered children.

## Materials and methods

### Animals

Adult male and female Wistar rats (Institute of Physiology, the Czech Academy of Sciences) were housed individually before mating in a temperature-controlled facility at 23 ± 2°C with free access to food and water. They were maintained under a light/dark cycle with 12 hours of light and 12 hours of darkness (LD12:12) with lights on and off at 06:00 and 18:00, respectively. The females were subjected to vaginal smears to determine stage of the estrous cycle phase. Mating was set on the night of pro-estrus and confirmed by presence of sperm in the vaginal smears on the next morning. In case of sperm positivity, this day was defined as day 0 of embryonic development (E0). The pregnant rats were weighted immediately after mating and then again before killing at E18 to E19 to calculate their body weight gain (with and without uterus), and a number of fetuses in the litters were recorded. The locomotor activity was monitored throughout the entire experiment (as described below).

All experiments were approved by the Animal Care and Use Committee of the Institute of Physiology (33/2019) and were performed in accordance with the Animal Protection Law of the Czech Republic as well as the European Community Council directives 86/609/EEC. All efforts were made to reduce the suffering of the animals.

### Experimental protocols

A total of 32 pregnant rats were used in the experiments. On day 7 of gestation, 16 rats were exposed to the sham surgery (Group A; transcriptomic study: *n* = 8, proteomic study: *n* = 8) and 16 rats to the surgical procedure for the SCN ablation (Group B; transcriptomic study: *n* = 8, proteomic study: *n* = 8); for details on the surgery procedure, see below. Group B included only rats with complete SCN lesion, as confirmed by the absence of locomotor rhythmicity in constant darkness and postmortem histological examination of the coronal brain sections throughout hypothalamic structures. Immediately after the surgery, both groups were released into constant darkness until the end of the experiment. Starting from E11, the rats of Group A continued in the ad libitum feeding regime, whereas the arrhythmic rats of the Group B had food access limited for only 8 hours a day (9:00 to 17:00) to impose group-synchronized feeding rhythmicity. The times of the onset of free-running locomotor activity (Group A) and the beginning of food-related activity (Group B) were assigned as the beginning of subjective night (CT12). The pregnant rats were killed under constant darkness in 3-hour intervals (1 rat per time point) to cover the 24-hour profile (CT0 to CT21) spanning fetal age approximately E18.5 to E19.5 (for clarity, we use E19 in the text). Fetal heads were frozen in dry ice, stored at −70°C, and then sectioned on Cryocut (Leica, Germany) for dissection of the SCN. For RNA-Seq, SCN from 8 fetuses of the same litter/time point were isolated by precooled microbiopsy punch, resulting in a cylindrical sample of SCN-containing frozen tissue with 0.3 mm diameter and approx. 0.4 mm height, then pooled together by immersion in lysis buffer and their RNA isolated on the same day. For proteomics, fetal SCNs were sampled and pooled in a similar way and kept at −70°C until protein extraction at mass spec facility.

### Surgical electrolytic ablation of the SCN

The bilateral SCN lesion was performed as previously described (2). The rats were anesthetized with isoflurane inhalation (Isoflurin, Vetpharma Animal Health, Spain) and mounted in a stereotactic instrument (David Kopf Instruments, Tujunga, CA, USA). The eyes were treated with eye drops Ophthalmo-Septonex (Zentiva, Czech Rep.). The electrode tips (0.2 mm diameter) were placed bilaterally into the SCN through drilled holes in the skull (coordinates: 1.3 mm caudal to bregma; ± 0.4 mm to midline; 9.2 mm below brain surface) and 1 mA current pulse was delivered for 20 seconds (53500 Lesion Making Device, Ugo Basile, Italy). The parameters were determined empirically to achieve complete lesion of the SCN and avoiding damage of surrounding tissue. Rats recovered in their home cages that were placed in constant darkness for the rest of the experiment. They received analgesic (NUROFEN Reckitt Benckiser Healthcare International, Great Britain) immediately after the surgery (applied per oral) and then in drinking water (2.5 ml per 250 ml) during the next day. The locomotor activity was monitored by infrared detectors. The success of the surgery was carefully checked behaviorally and histologically; rats with detectable rhythm in locomotor activity in constant darkness, and/or whose in which postmortem histological examination of the hypothalamic sections revealed presence of the SCN or its remaining parts, were excluded from the study. Sham surgery was performed to Group A in order to control for the plausible aftereffects of the surgical procedure on the pregnant rats and their fetuses. The sham-operated animals underwent the same procedure, including anesthesia and electrode insertion but without current application. None of the sham-operated rats exhibited disruption in circadian locomotor rhythmicity under constant darkness. For confirmation of the success of the surgery and intactness of the sham surgery, see Fig 1B.

### Locomotor activity and food intake measurement

The locomotor activity was monitored throughout the experiment. The rats were maintained individually in cages equipped with infrared movement detectors attached centrally above the top of each cage, and the activity was detected using a circadian activity monitoring system (Dr. H.M. Cooper, INSERM, France). The activity was recorded every minute and double-plotted activity records and periodograms were generated for visualization of the data. The parameters of circadian rhythmicity were analyzed using the ClockLab toolbox (Actimetrics, Illinois, USA) and Actiwatch Activity & Sleep Analysis V 5.42 software (Cambridge Neurotechnology, UK).

### RNA-Seq assay

Individual fetal SCN from 1 mother killed at each time point were pooled (*n* = 8 for Group A, *n* = 5 to 10 for Group B). RNA was isolated by RNeasy Micro Kit (Qiagen, Germany) according to manufacturer’s instructions, treated with On-Column DNAse I digestion set (Sigma) for 15 minutes at 37°C, then quantified by spectrophotometry. RNA integrity was tested by capillary electrophoresis (Agilent Tech., USA). Samples of purified total RNA (*n* = 16, 26 to 80 ng/μl, RIN > 9) were processed at Functional Genomics and Bioinformatics Service Laboratory of Institute of Molecular Genetics (Prague, Czechia) as follows: rRNA depletion, polyA RNA enrichment, and library preparation using Smarter Stranded low input tot-RNA-Seq kit v2 (Takara, Japan); sequencing using Illumina NextSeq 500 High output kit v2 (150 cycles, SE). Bioinformatics was performed using nf-core/rnaseq workflow pipeline (10.5281/zenodo.1400710). Reads were mapped to the latest UCSC rn6 (RGSC Rnor_6.0) rat genome assembly by HISAT2 v.2.1.0; read counts and FPKM were calculated by Salmon and Stringtie; all samples passed quality control. Before analysis, deseq2 was used to detect differential expression and to filter out less abundant transcripts—only transcripts with at least 1.5 raw count per million reads (up from default filter of 0.5/million) in at least 3 sample libraries were subsequently analyzed by dryR [33] and CompareRhythms [34] for detection of (differential) circadian expression patterns, clustering, and gene ontology. Normalized transcripts from both groups were categorized into 5 modules with a threshold of BICW > 0.5 based on their time-resolved expression pattern. Moreover, data were independently analyzed by Biocycle [48], eJTK [49], and MetaCycle [50].

### Enrichment analysis

Single- and multi-list pathway and process enrichment analysis was carried out on MetaScape [51] (GO Biological Process, Molecular Function and Cellular Component, KEGG, and Reactome pathways; R. norvegicus genome as background) for the subsets identified by dryR and CompareRhythms. Enriched terms (*P* < 0.01, min. gene count = 3, enrichment factor > 3) were grouped into clusters based on membership similarities (Kappa scores as similarity metric and sub-trees with Kappa > 0.3 considered as 1 cluster represented by the most statistically significant (GO) term). The 12 most statistically significant clusters of the GO Biological Process enrichment analysis of subsets identified by dryR (BICW > 0.5) were rendered as a network plot by Cytoscape 3.8.2. Additional enrichment analyses for each individual model were carried out on GOrilla, using arrhythmic and/or unassigned genes in as background, to confirm the MetaScape results.

### Proteomic assay

Samples (*n* = 16) of pooled fetal SCN were processed at Laboratory of Mass Spectrometry, Biocev (Prague, Czech Republic). Briefly, total proteins were extracted and digested, and peptides were subsequently analyzed on Orbitrap Fusion Tribrid Mass Spectrometer coupled with UltiMate 3000 RSLCnano liquid chromatography system (Thermo Fisher Scientific, USA). MaxQuant software package v.1.6.10.43 was used to gain final LFQ intensity values of 3,707 unique proteins in all samples; additional 183 peptides were assigned to 2 or more proteins and ca. 1,940 peptides showed undetectable LFQ in one or more samples; these were excluded from the analysis. Normalized data were then evaluated by Biocycle with *P* < 0.01 required to report potentially significant circadian change in protein levels across time points.

## Supporting information

S1 Fig Locomotor activity records. Recordings of locomotor activity (double-plotted actograms) of 8 pregnant rats from (A) Group A and (B) Group B. Rats were subjected to either sham surgery (Group A) or SCN lesion (Group B) on embryonic day E7 and then kept in constant darkness. Group A rats were fed ad libitum throughout the experiment and exhibited free-running locomotor activity (marked by yellow lines), the onset of which was determined circadian time 12. Group B rats were fed ad libitum during the interval between E9 and E11, when they were behaviorally arrhythmic (due to complete SCN ablation). From E11 until sampling at E18.5-E19.5, Group B rats were exposed to access to food restricted to 8 hours a day (tRF regime) (the timing of food availability is represented by red rectangles and the onset of food availability was assigned to circadian time 12). In most rats, the presence of food was accompanied by a slight increase in locomotor activity. SCN, suprachiasmatic nuclei; tRF, time-restricted feeding.
(DOCX)

S2 Fig Feeding activity. Distribution of average feeding activity of pregnant rats from Group A (red) and Group B (blue), respectively. Feeding patterns of Group A were analyzed manually via video recordings, while feeding activity of Group B was extrapolated from their locomotor activity records. As the analysis was carried out in constant darkness (DD), the endogenous circadian times of each individual were aligned, with subjective night (gray) based on the onset of activity (CT12-24, gray) and the (mostly) inactive state assigned subjective day (CT0-12, white).
(DOCX)

S3 Fig RNA-seq. Transcriptomic read counts were subjected to quality control before analysis. Deseq2 was used to detect differential expression and to filter out less abundant transcripts—only transcripts with at least 1.5 raw count per million reads (up from default filter of 0.5/million) in at least 3 sample libraries. (A) Total read counts. (B) Distribution of transformed data. (C) Density plot of transformed data. (D) Example of transcript levels correlation between 2 samples. Raw data have been deposited to NCBI’s Gene Expression Omnibus and are accessible through GEO Series accession no. GSE183172.
(DOCX)

S1 Data Maternal and fetal BWs and the size of the litter. Raw data for Fig 2. Naming of sheets corresponds to the relevant panels. BWs of 2 animals in Group B were not measured due to oversight. BW, body weight.
(XLSX)

S2 Data Clock gene transcripts analyzed by circadian rhythm detection methods. Eight different methods were used to evaluate rhythmicity of core clock genes—ARSER (ARS), JTK (JTK), Lomb-Scargle (LS), MetaCycle (meta2D), Biocycle, eJTK (from Biodare package), CompareRhythms, and dryR. *P* values and corresponding false discovery rate–adjusted *P* values, or BIC weights are described in corresponding columns.
(XLSX)

S3 Data dryR and CompareRhythms analyzis of RNA-Seq results. Amplitude (amp), relative amplitude (relamp), phase, model (no_rhythm, rhythmic in Group A *Arhy*, rhythmic in Group B *Brhy*, rhythmic in both groups with similar phase *ABsync*, rhythmic in both groups with different phase *ABdiff*), and BIC weight are shown in corresponding columns for both groups and both methods.
(XLSX)

S4 Data MetaScape analyzis results. dryR (DryR) and CompareRhythms (CR) results were analyzed by MetaScape; the output is in corresponding sheets. For details on MetaScape, see [51].
(XLSX)

S5 Data Nano LC-MS results and their Biocycle and CompareRhythms analyzis. Proteomic data (see sheet Annotation for raw label-free quantification LFQ values and their annotation) were analyzed by either Biocycle (results are in separate sheets for Groups A and B, LAG–phase identified by Biocycle), or by CompareRhythms (A_amp/B_amp–amplitude in group A/B).
(XLSX)

## Abbreviations

BICW: Bayes information criterion weight
FPKM: fragments per kb of transcript per million mapped reads
LFQ: label-free quantification
RT-qPCR: quantitative reverse transcription PCR
SCN: suprachiasmatic nuclei
tRF: time-restricted feeding
