# Supplementary material for: Early rhythmicity in the fetal suprachiasmatic nuclei in response to maternal signals detected by omics approach
Source: PLoS Biol. 2022 May 24;20(5):e3001637. doi: 10.1371/journal.pbio.3001637 (PMC9129005; doi:10.1371/journal.pbio.3001637)
Supplement: S1 Fig — Recordings of locomotor activity (double-plotted actograms) of 8 pregnant rats from (A) Group A and (B) Group B. Rats were subjected to either sham surgery (Group A) or SCN lesion (Group B) on embryonic day E7 and then kept in constant darkness. Group A rats were fed ad libitum throughout the experiment and exhibited free-running locomotor activity (marked by yellow lines), the onset of which was determined circadian time 12. Group B rats were fed ad libitum during the interval between E9 and E11, when they were behaviorally arrhythmic (due to complete SCN ablation). From E11 until sampling at E18.5-E19.5, Group B rats were exposed to access to food restricted to 8 hours a day (tRF regime) (the timing of food availability is represented by red rectangles and the onset of food availability was assigned to circadian time 12). In most rats, the presence of food was accompanied by a slight increase in locomotor activity. SCN, suprachiasmatic nuclei; tRF, time-restricted feeding. (DOCX) [file pbio.3001637.s001.docx]

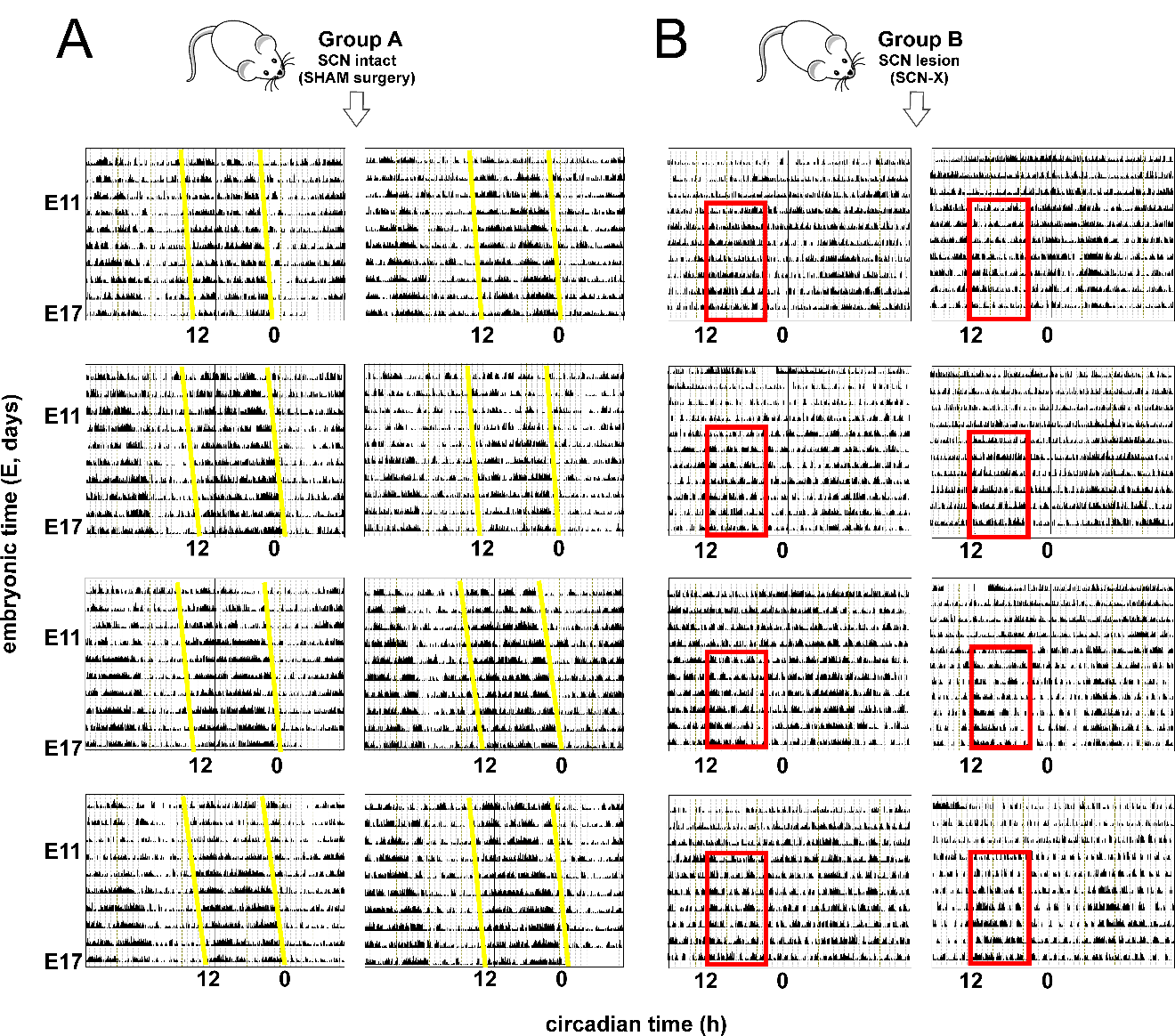


**S1 Fig.** **Locomotor activity records.** Recordings of locomotor activity (double-plotted actograms) of 8 pregnant rats from (A) group A and (B) group B. Rats were subjected to either sham surgery (group A) or SCN lesion (group B) on embryonic day E7 and then kept in constant darkness. Group A rats were fed ad libitum throughout the experiment and exhibited free-running locomotor activity (marked by yellow lines), the onset of which was determined circadian time 12. Group B rats were fed ad libitum during the interval between E9 and E11, when they were behaviorally arrhythmic (due to complete SCN ablation). From E11 until sampling at E18.5-E19.5, group B rats were exposed to access to food restricted to 8 h a day (time-restricted feeding regime, tRF) (the timing of food availability is represented by red rectangles and the onset of food availability was assigned to circadian time 12). In most rats, the presence of food was accompanied by a slight increase in locomotor activity.
