# Supplementary material for: Early rhythmicity in the fetal suprachiasmatic nuclei in response to maternal signals detected by omics approach
Source: PLoS Biol. 2022 May 24;20(5):e3001637. doi: 10.1371/journal.pbio.3001637 (PMC9129005; doi:10.1371/journal.pbio.3001637)
Supplement: S2 Fig — Distribution of average feeding activity of pregnant rats from Group A (red) and Group B (blue), respectively. Feeding patterns of Group A were analyzed manually via video recordings, while feeding activity of Group B was extrapolated from their locomotor activity records. As the analysis was carried out in constant darkness (DD), the endogenous circadian times of each individual were aligned, with subjective night (gray) based on the onset of activity (CT12-24, gray) and the (mostly) inactive state assigned subjective day (CT0-12, white). (DOCX) [file pbio.3001637.s002.docx]

**
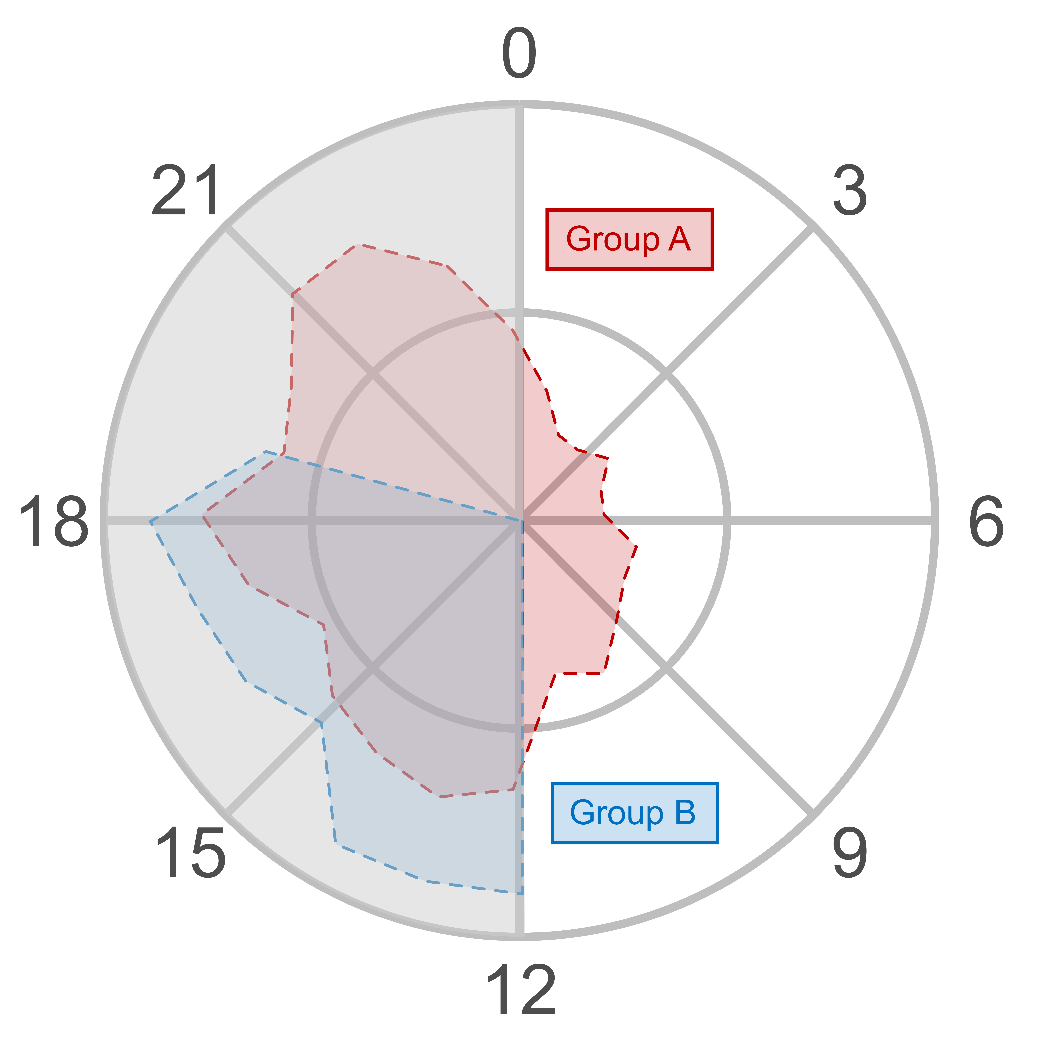
**

**S2 Fig.** Distribution of average feeding activity of pregnant rats from Group A (red) and Group B (blue), respectively. Feeding patterns of Group A were analyzed manually via video recordings, while feeding activity of Group B was extrapolated from their locomotor activity records. As the analysis was carried out in constant darkness (DD), the endogenous circadian times of each individual were aligned, with subjective night (gray) based on the onset of activity (CT12-24, gray) and the (mostly) inactive state assigned subjective day (CT0-12, white).
