# Supplementary material for: Early rhythmicity in the fetal suprachiasmatic nuclei in response to maternal signals detected by omics approach
Source: PLoS Biol. 2022 May 24;20(5):e3001637. doi: 10.1371/journal.pbio.3001637 (PMC9129005; doi:10.1371/journal.pbio.3001637)
Supplement: S3 Fig — Transcriptomic read counts were subjected to quality control before analysis. Deseq2 was used to detect differential expression and to filter out less abundant transcripts—only transcripts with at least 1.5 raw count per million reads (up from default filter of 0.5/million) in at least 3 sample libraries. (A) Total read counts. (B) Distribution of transformed data. (C) Density plot of transformed data. (D) Example of transcript levels correlation between 2 samples. Raw data have been deposited to NCBI’s Gene Expression Omnibus and are accessible through GEO Series accession no. GSE183172. (DOCX) [file pbio.3001637.s003.docx]

**
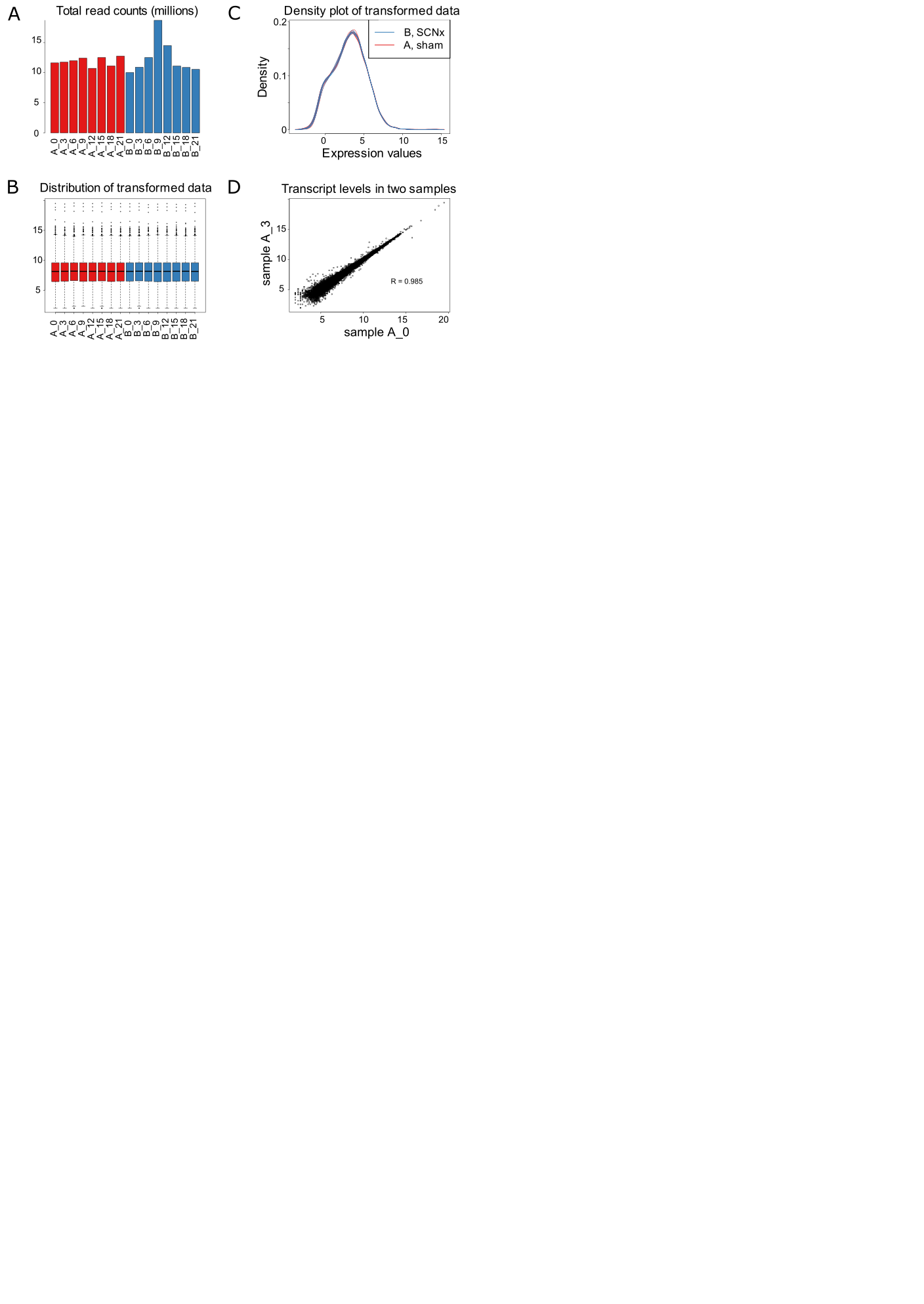
**

**S3 Fig.** Transcriptomic read counts were subjected to quality control before analysis. Deseq2 was used to detect differential expression and to filter out less abundant transcripts – only transcripts with at least 1.5 raw count per million reads (up from default filter of 0.5/million) in at least 3 sample libraries. **(A)** Total read counts. **(B)** Distribution of transformed data. **(C)** Density plot of transformed data. **(D)** Example of Transcript levels correlation between two samples. Raw data have been deposited to NCBI’s Gene Expression Omnibus and are accessible through GEO Series accession no. GSE183172.
